# Estimating postoperative survival rate of gastric cancer patients and its effective factors in Babol, northern Iran: 2006-2011

**DOI:** 10.22088/cjim.8.3.190

**Published:** 2017

**Authors:** Seyed-Reza Modarres, Abdolrahim Gholizadeh-Pasha, Soheil Khatibi, Sepideh Siadati

**Affiliations:** 1 Department of Surgery, Faculty of Medicine, Babol University of Medical Sciences, Babol, Iran.; 2 Babol University of Medical Sciences, Babol, Iran.; 3 Cancer Research Center, Health Research Institute, Babol University of medical Sciences, Babol, Iran.; 4 Department of pathology, Faculty of Medicine, Babol University of Medical Sciences, Babol, Iran.

**Keywords:** Gastric cancer, Survival, Tumor stage

## Abstract

**Background:**

The importance of gastric cancer, considering its progressive course and high mortality is one of the reasons we pay attention to patients’ survival and the significance of this study was to estimate survival and determine the effective factors on patients with gastric cancer.

**Methods:**

In this cross-sectional study, all patients with gastric cancer who underwent surgery in Shahid Beheshti Hospital, Babol, northern Iran during 2006-2011 were enrolled. To get information, a checklist was prepared consisting gender, age, and other factors related to survival from the start of diagnosis to the end of study. Statistical analysis was performed using SPSS Version 20, t-test, chi-square test and Cox regression and Kaplan-Meier.

**Results:**

One hundred seventy-five patients consisting 132(75.4%) males and 43(24.6%) females entered the study. Among the 146 patients who were followed up for 50 months, 25 (14.28%) cases survived and 121 (69.15%) cases died. Survival did not have a significant association with gender, age, family history, smoking, location and type of tumor, metastasis, involved lymph nodes and treatment. Tumor stage and type of surgery had a significant association with survival in Cox regression.

**Conclusion:**

Diagnosis of cancer at the early stage and type of surgery increased survival rate

**D**yspepsia, peptic ulcer, reflux, gastric bleeding, gastritis due to Helicobacter pylori and gastric cancer are the common diseases of the stomach as the second part of digestive tract. Gastric cancer is the uncontrolled growth of malignant cells in which 85% are adenocarcinoma and 15% are lymphoma and gastro intestinal stromal tumor (1-3). Gastric cancer is the second most common cancer in the world and according to statistics of Iran Cancer Research Center, it is the most common cancer in men and the third common cancer in women after breast and colon cancer (4). Despite some improvements in the treatment, a 5-year survival rate is still low (5). Estimating cancer patients survival is very important. Being a male, age more than 40, family history of gastric cancer, chronic gastritis, high protein diet with lack of fruits and vegetables intake are the predisposing factors (1). The symptoms of this disease include: weight loss, lack of appetite, early satiety, nausea and vomiting (5-7). Any delay in the diagnosis of gastric cancer leads to an increase in the possibility of metastasis (1). To determine metastasis and preoperative staging, computed tomography scan (CT scan), positron emission tomography scan (PET scan) or endosonography have been used. The main treatment of gastric cancer is surgery accompanied by radiotherapy and chemotherapy (5).

In the present study, we studied some factors affecting survival and compare them with other studies.

## Methods

This cross-sectional study was conducted on all patients with gastric cancer who underwent surgery at Shahid Beheshti Hospital in Babol, northern Iran during 2006-2011. Informed consent was obtained and the study was approved by the Ethics Committee of Babol University of Medical Sciences. Those patients under 20 years old and those patients with other types of cancer in addition to gastric cancer were excluded. To collect information, a checklist was prepared which include gender, family history of gastric cancer, smoking, location of cancer determined by endoscopy, type of surgery, type of cancer, metastasis, involved lymph nodes, postoperative TNM staging based on pathological records (ICD-0), the length of hospital stay, chemotherapy and/or radiotherapy, survival from the time of diagnosis to the end of study. The information was obtained from the patient’s hospital file plus via phone call. Patients who did not answer were excluded from the study. The data were analyzed by SPSS Version 20, and t-test, chi-square, Kaplan-Meier, Cox regression. The significant p-value considered was less than 0.05.

## Results

**Figure 1:**
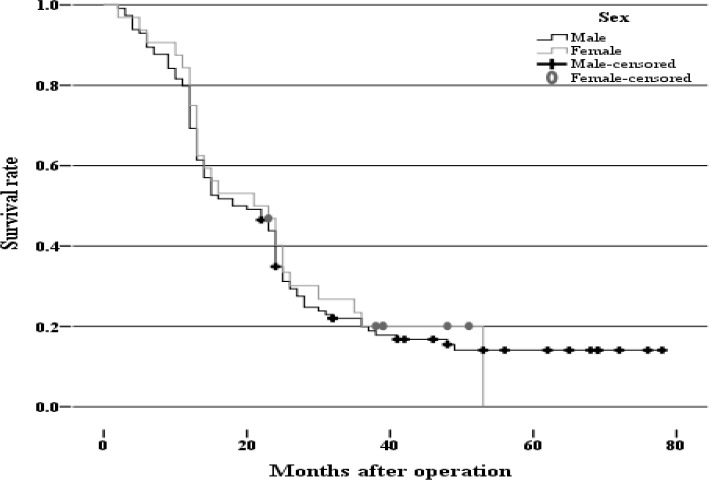
The possible survival of patients with gastric cancer during 2006-2011

**Figure 2:**
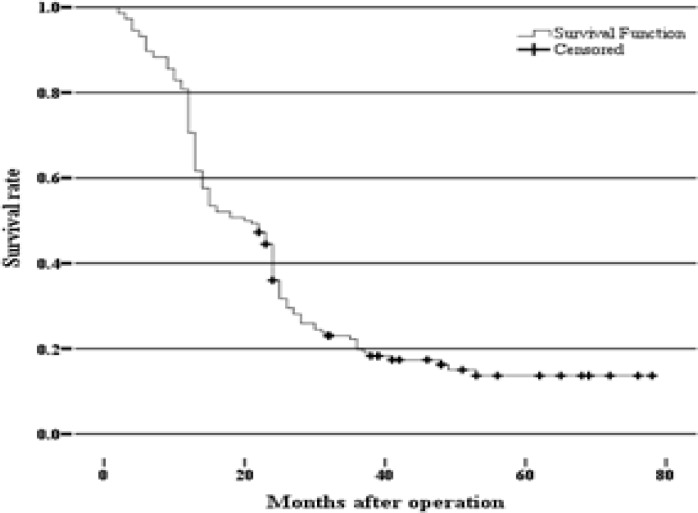
The possible survival of patients with gastric cancer according to gender in 2006-2011

One hundred seventy-five gastric cancer patients that consist of 132 (75.4%) males and 43(24.6%) females, entered the study during 2006-2011. The mean age for men was 65.84±13.00 years and for women was 58.48±13.09 years. The most prevalent age range was 61-75 years (table 1). One hundred forty-six patients were followed–up at a mean of 50 months, and of these 25 (14.3%) patients survived and 121 (69.2%) patients died. The average survival was 26.9 months with 95% confidence interval (figure 1) and there was no association between survival and gender (figure 2).

**Table 1:** Distribution of age in patients with gastric cancer during 2006-2011

**Range of age(yrs.)**	**No. (%)**
45≥	18(12.3)
46-60	24(16.4)
61-75	68(46.6)
76≤	36(24.7)

The relatives of 18 (10.3%) patients suffered from cancer which include 10 stomach, 3 cardia, 4 esophagus and one laryngyal carcinoma with 2.68 (0.98-7.34) OR with 95% confidence interval. Twenty-six (17.8%) and 120 (82.21) cases were smokers and nosmokers, respectively. 103 (70.5%) patients had preoperative endoscopy and proximal of stomach was the most involved location (48.6%). Total gastrectomy (58.9%) was the most common type of surgery. Based on pathology results, adenocarcinoma (89.0%) was the most common type of cancer. There was metastasis in 42 (33.1%) patients, while 135 (77.1%) patients, lymph nodes were involved. Stage IIIA (27.4%) was the most common and Stage IA (2.9%) was the least common stage. The duration of hospitalization varied from 1 to 17 days (9.12±2.35). Thirty-five (20%) patients had chemotherapy and 9(5.1%) had chemotherapy accompanied by radiotherapy (table 2). 

**Table 2:** The effective factors of survival in patients after surgery by Cox regression

**Variable**	**Hazard Ratio**	**Confidence Interval 95%**	**Pvalue**
**Gender**			
Men Women	1 0.95	1 0.59-1.52	0.83
**Age**			
45≥ 46-60 61-75 76≤	1 0.47 0.72 0.58	1 0.22-1 0.38-1.35 0.28-1.20	0.06 0.31 0.14
**Family history**			
Yes No	1 0.61	1 0.30-1.23	0.17
**Smoking**			
Yes No	1 0.86	1 0.50-1.49	0.6
**Tumor location**			
Proximal Middle Distal Two-third or more	1 1.23 0.82 0.67	1 0.76-1.99 0.44-1.52 0.35-1.27	0.39 0.54 0.22
**Type of tumor**			
Adenocarcinoma Signet cell carcinoma	1 1.73	1 0.80-3.76	0.16
**Metastasis**			
Yes No	1 0.64	1 0.39-1.04	0.07
**Metastasis level**			
Serosa Nerve and vascular trunk Lipid Muscular Serosa and lipid	1 0.93 1.82 1.41 1.08	1 0.47-1.83 0.77-4.28 0.80-2.48 0.53-2.18	0.85 0.16 0.22 0.81
**Lymph nodes**			
Yes No	1 0.97	1 0.63-1.49	0.9
**Tumor stage**			
IA IB IIA IIIA IIIB IV	1 0.75 3.55 5.37 8.78 6.31	1 0.17-3.31 0.72-17.34 0.94-30.46 1.45-52.97 1.15-34.71	0.71 0.11 0.6 0.01 0.03
**Chemotherapy**			
Yes No	1 0.91	1 0.51-1.60	0.74
**Radiotherapy with chemotherapy**			
Yes No	1 0.96	1 0.34-2.72	0.93
**Type of surgery**			
Total gastrectomy Hemi gastrectomy Subtotal gastrectomy Proximal gastrectomy Non-removal	1 0.26 0.42 0.59 0.13	1 0.12-0.57 0.18-0.97 0.25-1.14 0.01-1.14	0.001 0.04 0.23 0.06

## Discussion

Gastric cancer is the fourth most common cancer and the second leading cause of cancer related deaths (7). Early diagnosis and considering other factors like age, gender, positive family history and stage help in appropriate therapeutic management and increase survival.

Zeinalzadeh Chinibelagh in 2012 studied the age pattern of the different types of cancer in Eastern Azerbijan. The mean age of gastric cancer patients was 65±13.5 years that was similar to our study (9). Some studies declared that increasing age leads to decreasing survival, however, in our study we could not find any relation. It was shown in Yazdanpanah’s study in Ardebil that age as well as the stage, positive family history, alocohol drinking and smoking have no effect on the 4-year survival of the patients with upper gastrointestinal cancers (10). Nonetheless, in the current study, the end stage patients had six times lower survival rate. Zeraati et al. showed 23.6% survival rate (19.90 months). In this study, gender, location and type of tumor had no significant effect; although, stage and metastasis had significant effect on the survival (p<0.0001) (11). In our study, stage had significant association with survival rate, yet, no association was seen with metastasis. The different ratios of genders in various studies especially the few patients in our study could be considered for this difference. Safaii et al. concluded that those with at least one family member with gastric cancer were 3 times more at risk for gastric cancer. Of the 746 patients with gastric cancer, 117 patients had first-degree relatives involved with gastric cancer and 71 patients had second-degree relatives with gastric cancer (12). 

Family history of cancer has already been mentioned as a risk factor, nonetheless, in our study, no association was seen with survival. The increased risk of having cancer is 10 times more in people with cancer history among the first-degree relatives (13). Which might be due to genetic factors and similar lifestyle, especially diet. Because of the low number of patients in this study and also positive family history, no significant association between family history and survival was obtained. The findings of studies by Yazdanband et al. and Veisani et al. showed that the estimated survival rate of patients with gastric cancer and the associated factors in Sanandaj, Iran were similar to our study (10, 14).

In this study, survival rate had no association with the type of tumor. There was not a significant association between the type of tumor and improvement rate in Atoof et al’s. study (15). Moreover they showed no significant association between the site of tumor, metastasis, site of metastasis, and stage with survival. Ehich was similar to our study. Akhavan et al. also studied the survival rate of patients with gastric adenocarcinoma in Yazd, Iran. Two-year survival was 50% and three- year survival was 30% with 29.97 mean survival rate. There was a significant association between the stage, therapeutic management and metastasis with survival, but not with the subtype of adenocarcinoma, location and grade of tumor and type of chemotherapy that was similar to our study (16). Zhang et al. studied the predicting factor on survival in patients with proximal gastric cancer, and stated that the presence of invasion, positive lymph node and metastasis are effective factors (18). In the current study, metastasis and the different stages of invasion had no effect on survival. 

Zeraati et al., showed that the number of involved lymph nodes and the type of surgery were effective on the survival of patients (19). Besides, Zhang concluded that the type and size of tumor, types of surgery, positive surgical margin, lymphovascular invasion and stage were not the predictive factors for survival. Nonetheless, invasion, poor differentiation, number of obtained lymph nodes, positive lymph nodes and distant metastasis have effect on the survival of the patients (18). In the current study, no significant association between the involved lymph nodes and survival was seen. 

Wang et al. showed the most and the least common frequency of gastric cancer were in stage III and I, respectively (19). In Roshanai’s study on 262 gastric cancer patients in Tehran, Iran showed, stage II had the minimum frequency in 59 (22.5%) patients and stage III had the maximum frequency 125 (47.7%) (3). In our study, stages III A and IA had the minimum and maximum frequencies respectively. Delay in the diagnosis of gastric cancer due to its similarity in symptoms with other gastrointestinal diseases, could explain this difference. Maroofizadeh et al. showed the significant association between the patient’s age size of tumor and survival stage. This was done by analyzing the associated factors for survival in patients with gastric cancer using additive hazards model (20). Roshanai also showed the pathologic stage of gastric carcinoma as an effective factor in the patient’s survival rate (5). Although this significant association was not seen in our study. This could be due to the large number of patients with higher stages of cancer. Kadowaki et al. in a study showed that the patients with appropriate chemotherapy and only one metastasis may survive longer than those who did not receive efficient and appropriate chemotherapy with several metastases (17). However, in our study, chemotherapy alone and chemotherapy with radiotherapy did not have significant association with survival we considered the reason might be due to poor cooperation of patients, especially with no information about the type of treatment.

One of the limitations of this study was the small sample size, especially those patients in the stage I. Most patients were diagnosed in higher stages, therefore, survival rate is very low. Although there have been possible confounding variables due to collection of retrospective data. 

In conclusion according to the findings of this study and the rather low survival rate of gastric cancer patients, screening programs are mandatory for early diagnosis. It has been recommended to pay more attention to preventive programs by giving information to the population about risk factors.
